# Improved survival following surgery and radiation therapy for olfactory neuroblastoma: analysis of the SEER database

**DOI:** 10.1186/1748-717X-6-41

**Published:** 2011-04-25

**Authors:** Mary E Platek, Mihai Merzianu, Terry L Mashtare, Saurin R Popat, Nestor R Rigual, Graham W Warren, Anurag K Singh

**Affiliations:** 1 Division of Cancer Prevention and Population Sciences, Roswell Park Cancer Institute, Buffalo, New York, USA; 2 Department of Pathology, Roswell Park Cancer Institute, Buffalo, New York, USA; 3 Department of Biostatistics, Roswell Park Cancer Institute, Buffalo, New York, USA; 4 Department of Head and Neck/Plastic Surgery, Roswell Park Cancer Institute, Buffalo, New York, USA; 5 Department of Radiation Medicine, Roswell Park Cancer Institute, Buffalo, New York, USA

## Abstract

**Background:**

Olfactory Neuroblastoma is a rare malignant tumor of the olfactory tract. Reports in the literature comparing treatment modalities for this tumor are limited.

**Methods:**

The SEER database (1973-2006) was queried by diagnosis code to identify patients with Olfactory Neuroblastoma. Kaplan-Meier was used to estimate survival distributions based on treatment modality. Differences in survival distributions were determined by the log-rank test. A Cox multiple regression analysis was then performed using treatment, race, SEER historic stage, sex, age at diagnosis, year at diagnosis and SEER geographic registry.

**Results:**

A total of 511 Olfactory Neuroblastoma cases were reported. Five year overall survival, stratified by treatment modality was: 73% for surgery with radiotherapy, 68% for surgery only, 35% for radiotherapy only, and 26% for neither surgery nor radiotherapy. There was a significant difference in overall survival between the four treatment groups (p < 0.01). At ten years, overall survival stratified by treatment modality and stage, there was no significant improvement in survival with the addition of radiation to surgery.

**Conclusions:**

Best survival results were obtained for surgery with radiotherapy.

## Background

Olfactory neuroblastoma (ONB) or esthesioneuroblastoma is an uncommon neuroendocrine malignancy which was first described by Berger et al. in 1924 [1]. ONB accounts for approximately 3% of endonasal neoplasms [2]. Though the etiology is unknown[2], ONB appears to arise from the olfactory membrane of the sinonasal tract and preferentially involves the anatomic distribution of the epithelium overlying the cribriform plate[2], superior turbinate and the superior nasal septum [3]. Patients most commonly present with nonspecific symptoms of nasal obstruction and epistaxis [2]. Less common symptoms include headache, pain, visual disturbances and anosmia [2]. ONB affects both sexes equally with a bimodal age distribution (the 2^nd ^and 6^th ^decades of life) although patients of all ages can be affected [2].

The rarity of ONB has limited study to individual case reports [4-8], small series [9-15], meta-analysis of such small series[16], or registry reports [17] and precluded prospective trials. Based on such limited data, the gold standard of care for these tumors is craniofacial resection followed by adjuvant radiotherapy [10,18,19].

The Surveillance, Epidemiology, and End Results (SEER) database, which collects cancer incidence and survival data from cancer registries that are population-based and cover approximately 26% of the United States population [20], was used to identify a large series of patients with ONB. In a prior report of the SEER database, Jethanamest et al. were unable to show a significant improvement in overall survival with the addition of radiation to surgery [17]. The objective of this study was to re-assess survival outcomes between different treatment modalities among the ONB cases identified from the SEER database with an additional four years of data.

## Methods

### Identification of Cases

The SEER database for the years 1973 to 2006 was used to examine management strategies for ONB. The diagnosis code of 9522/3 was queried and all records were found in the following sties: C30.0 (nasal cavity), C31.0 (maxillary sinus), C31.1 (ethmoid sinus), C31.2 (frontal sinus), C31.3 (sphenoid sinus), C31.8 (overlapping lesion of accessory sinuses) and C31.9 (accessory sinus, NOS). Information for the following treatment groups was queried: both surgery and radiotherapy, surgery only, radiotherapy only, neither surgery nor radiotherapy. Staging in the SEER data is based on classification criteria that vary by site and year of diagnosis. The SEER historic staging variable provided information for the following categories: localized, regional, distant and unstaged. Information for type, timing, and duration of chemotherapy was not available from the SEER database. The neuroepithelioma code, 9523/3, was also queried but there were no cases identified using this code.

### Statistical Analysis

Treatment group information was summarized using frequencies and cumulative frequencies. The Kaplan-Meier method was used to estimate overall survival distributions by treatment modality. A Cox multiple regression analysis was performed using treatment, race, SEER historic stage, sex, age at diagnosis, year at diagnosis and SEER geographic registry. Overall survival was then estimated by stage comparing surgery only versus surgery with radiotherapy for local stage, regional stage and for local plus regional. These analyses were truncated for anyone with overall survival greater than 10 years. Statistical assessment of observed differences in survival distributions was done using the log-rank test in conjunction with a Bonferroni adjustment for multiple comparisons. A 0.05 nominal significance level was used in all hypothesis testing. Data analyses were performed using SAS, version 9.1.3, statistical software (SAS Institute., Cary, NC).

## Results

A total of 511 cases of ONB were reported for the years 1973 to 2006. A description of this cohort can be found in Table 1. There was a unimodal distribution of ages with most cases between the ages of 40 and 70 years old (mean age was 53 years, SD of 18). The majority of cases were treated with both surgery and radiotherapy (61%), were white with 55% male and a primary tumor site in the nasal cavity. Information concerning whether radiation therapy was administered before or after surgery was not available. Approximately 22% of the cases received surgery only, 11% received radiation therapy alone and approximately 6% did not receive surgery or radiotherapy. The distribution of treatment methods and outcomes among the 511 cases is shown in Table 2. There was a statistically significant difference in the overall survival between these four treatment groups (surgery and radiotherapy, surgery only, radiotherapy only, and neither surgery nor radiotherapy) (p < 0.01). The percentage of cases surviving five years by treatment modality was: 73% for surgery and radiotherapy, 68% for surgery only, 35% for radiotherapy only and 26% for neither radiotherapy nor surgery.

**Table 1:** Description of SEER ONB cohort (N = 511)

Characteristic	Frequency	Percent
**Age (years)**		
**(n = 485)**		
**<25**	38	8
**25-39**	59	12
**40-69**	299	62
**>70**	89	18

**Gender**		
**Male**	283	55
**Female**	228	45

**Race**		
**White**	415	81
**Black**	41	8
**Other**	51	10
**Unknown**	4	1

**Primary Tumor Site**		
**Nasal Cavity**	370	72
**Maxillary sinus**	22	4
**Ethmoid Sinus**	65	13
**OthSinus (frontal, sphenoid accessory sinuses)**	54	11

**SEER Historic Stage**		
**(n = 473)**		
**Localized**	112	24
**Regional**	210	44
**Distant**	129	27
**Unstaged**	22	5

**Treatment**		
**(n = 485)**		
**Both Surgery and Radiation**	296	61
**Surgery Only**	105	22
**Radiation Only**	53	11
**Neither Surgery Nor Radiation**	31	6

**Table 2:** Distribution of Treatment Methods and Outcomes for SEER ONB

Treatment	Total n = 485* N (%)	Number Failed	Number Censored	Median Estimate (months)
**Both Surgery and Radiotherapy**	296 (61.0)	109	187	150

**Surgery Only**	105 (21.7)	41	64	169

**Radiotherapy Only**	53 (10.9)	30	23	39

**Neither Surgery nor Radiotherapy**	31 (6.4)	18	13	10

*26 cases with either surgery and/or radiotherapy information missing

**Figure 1:**
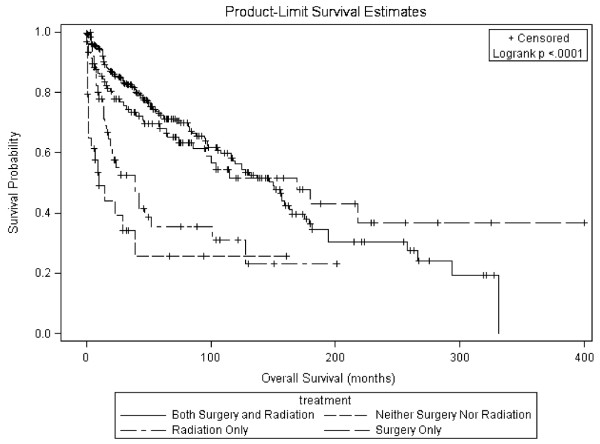
**Overall Survival of SEER ONB Cases by Treatment Groups**.

Pair-wise comparisons demonstrated a statistically significant difference in the overall survival distributions between four of the pairs. The overall survival distribution between patients who received both surgery and radiotherapy was significantly different from patients who received radiotherapy only (p < 0.01) and from patients who received neither surgery nor radiotherapy (p < 0.01). Additionally, the overall survival distribution between patients who received surgery only was significantly different from patients who received neither surgery nor radiotherapy (p = 0.03) and from patients who received radiotherapy only (p = 0.046) All other comparisons were not significantly different. Figure 1 shows overall survival curves stratified by treatment groups.

Cox multiple regression analysis was performed to adjust for interaction between covariates and confirmed that treatment remained a significant predictor of overall survival (p < 0.01). However, the overall survival distribution between patients who received surgery only was no longer significantly different from patients who received radiotherapy only (p = 0.13).

Estimated ten year overall survival comparing surgery only versus surgery with radiotherapy stratified by local stage, regional stage and local plus regional stage showed no difference between these treatment methods for any stage.

## Discussion

This analysis of the SEER database represents the largest published series of ONB cases. Five year overall survival stratified by treatment was: 73% for surgery and radiotherapy, 68% for surgery only, 35% for radiotherapy only, and 26% for neither surgery nor radiotherapy. There was a significant difference in overall survival between the four treatment groups (p < 0.01). There was a significant difference in five year overall survival distributions between patients receiving surgery and radiotherapy and patients receiving radiotherapy only (p < 0.01).

Of note, only 31 patients received "neither surgery nor radiotherapy." Based on historic staging information from the SEER database, 84% of these patients had regional or distant disease. The lack of a significant survival benefit compared with surgery alone or surgery with radiotherapy is likely an artifact of these small numbers.

Based on five year survival stratified by treatment and consistent with one meta-analysis, smaller series and a previous examination of the SEER database, this analysis of SEER data shows that surgery with radiotherapy provides optimal management for ONB [9,10,16,17]. Jethanamest et al. analyzed the SEER database from 1973-2002, identified 311 eligible patients, and reported longest duration of mean survival was for cases receiving both surgery and radiotherapy but also reported that the only significant differences between treatment groups was for those receiving radiotherapy alone and those receiving combined modality treatment [17]. The authors also performed a detailed analysis in which they attempted to infer the patients' Kadish stage from the information available in the SEER database. Such an analysis, lacking any clinical or radiologic basis, has obvious limitations which were ably enumerated by the authors in their discussion. Due to these limitations, we did not make an effort to repeat inference of the Kadish stage.

Dulguerov et al. performed a meta-analysis of ONB publications between 1990 and 2000 (26 studies, 390 patients) with the objective to review recent developments in diagnosis, staging and treatment [16]. The optimal approach to treatment in this meta-analysis was a combination of surgery and radiotherapy. Gruber et al. and Lund et al. concluded the same (Table 3) [9,10].

**Table 3:** Summary of Published Single Institution Experiences

Study	Year	Institution	Period	No. of patients (f/u in months)	Treatment Received	Findings
**Gruber et al. **[9]	2002	Universities of Berne and Zurich	1980-2001	28 (68)	Group 1: S (radical) + RT Group 2: S(partial) + RT	10 year DFS: Group 1: 55% Group 2: 0%

**Lund et al. **[10]	2003	University College London	1978-2001	45 (57)	Group 1: S alone Group 2: S + RT	Local Recurrence Group 1: 28% Group 2: 8%

Studies Including Chemotherapy						

**Rastogi et al. **[11]	2006	King George Medical University	1988-2004	8 (36)	All Patients: S (NCFR) + RT + C	3 year DFS/OS: 72.9%/71.4%

**Kim et al. **[12]	2007	Four General Hospitals in South Korea	1990-2004	10 (44.9)	Group 1: S (+/- RT) Group 2: CCRT	5 year DFS: Group 1: 68% Group 2: 42%

**McLean et al. **[13]	2007	Emory University-affiliated hospitals	1991-2006	21 (47)	Group 1: S Group 2: S + RT Group 3: S +RT+C	Local Recurrence: Group 1: 0% Group 2: 53.3% Group 3: 43%

**Porter et al. **[14]	2008	Mayo Clinic Rochester	1976-2003	12 (N/A)	Group 1: S (+/- RT) Group 2: S + C (+/- RT)	Median OS: Group 1: 78 months Group 2: 83+ months

**Bachar et al. **[15]	2008	Princess Margaret Hospital	1972-2006	39 (82.3)	Group 1: S Group 2: C Group 3: RT Group 4: S + RT	10 year OS: 69.2% (all groups; OS per group N/A) • S +RT optimal • C did not influence outcome

**Abbreviations: **f/u (follow-up), S (surgery), RT (radiotherapy), DFS (disease free survival), NCFR (non-craniofacial resection), C (chemotherapy), OS (overall survival), CCRT (concurrent chemoradiotherapy), N/A (not available)

Radical surgery, however, of early stage lesions is not performed at all centers [4], and there are reports that endonasal endoscopic resection and postoperative adjuvant radiotherapy yields comparable outcomes to open craniofacial resection and adjuvant radiation therapy [18,21-23]. The advantage of sample size in this SEER analysis, while a distinct benefit in comparison to smaller single institution series, does come at the cost of limited documentation of treatment detail. For example, the SEER database did not include type of surgical resection.

In a recent meta-analysis of patient data for ONB between 1992 and 2008, endoscopic surgery was shown to be a valid treatment method to open surgery [24]. This SEER analysis is not able to discriminate any potential differences in outcomes with open craniofacial versus endoscopic resection. Additionally, the time period for the SEER database, 1973-2006, includes a time period before the beginning of the modern age of skull base surgery (1985-1990). A stratification of patient survival by year of diagnosis may facilitate understanding if current treatment paradigms are better than prior ones and particularly for surgical procedure, but the limited numbers even in this cohort would make any conclusions based on stratified analyses untenable. We did include year at diagnosis as a covariate in our Cox regression model.

### The Role of Chemotherapy

The SEER database did not include information for those treated with chemotherapy, however recent reports of outcomes for ONB patients have included chemotherapy [11-15]. These findings are summarized in Table 3.

### Selection Bias: A Major Limitation of Any Retrospective Review

Selection bias, which confounds any retrospective review, is particularly relevant in this analysis when comparing the surgery only and surgery plus radiation groups. This bias exists because clinicians treating the patients whose data is captured in the SEER database made decisions to give or omit radiation following surgery often based on clinical/pathologic/radiographic information that is not captured by SEER. Thus, one suspects that patients given surgery and radiation had poorer prognostic factors than those who received surgery alone. The absence of a statistically significant survival benefit with the addition of radiation must be interpreted within the context of this possible selection bias. In this analysis, we examined ten year survival by stage (local, regional and local plus regional) for surgery compared to surgery with radiotherapy and did not find a statistically significant difference for any stage.

### Other Limitations of the SEER Database: Pathologic, Terminology, Taxonomy, Grading and Staging Considerations

ONB is a tumor restricted to the area of olfactory neuroepithelium, which arises from embryonic olfactory placodes and in adults is replaced partially by respiratory mucosa. Not surprisingly in a tumor arising from the neural-epithelial olfactory mucosa, the phenotype of ONB is intermediate between that of pure neural neoplasms (neuroblastoma and paraganglioma) and neuroendocrine epithelial tumors (carcinoid, neuroendocrine carcinoma, small cell carcinoma) [25]. This intrinsic heterogeneity accounts for the various synonyms used to describe this neoplasm in the past: esthesioneuroblastoma, esthesioneuroepithelioma, (esthesio)neurocytoma, and even neuroendocrine carcinoma [26]. Due to its anatomic location, ONB is diagnosed in clinical practice by general surgical/head and neck pathologists and/or neuropathologists (depending on the surgical approach) who might use a slightly different terminology, as illustrated by the respective ONB description in the two corresponding WHO tumor classifications [2,25].

In addition, pathologic ONB definition has been refined in the period studied from being based exclusively on histomorphology [27-29] to include ultramicroscopic findings [26,29-33] and immunoprofile[2,25,32,34-36] in the diagnostic process. Regardless, it is worth noting that most ONB occurring in their characteristic location are easily recognizable, especially on the low grade side of the spectrum, which account for most cases reported in other series and (presumably) for the tumors reported to SEER database.

The only grading system available for ONB is unchanged from 1988 when Hyams proposed it based on the Armed Forces Institute of Pathology experience [37]. This system divides the ONB into four grades ranging from well (I) to least differentiated (IV) based on the tumor architecture, cellular pleomorphism, presence of neurofibrillary matrix and rosettes, mitotic activity, and presence of necrosis or calcifications. The grading system is somewhat subjective and sampling dependent (absent a complete resection) and its reproducibility is difficult to estimate due to the rarity of this disease.

The clinical staging introduced by Kadish a decade earlier describes mostly tumors of low Hyams grade [28]. A revised Kadish staging system which describes a stage D tumor as consisting of cervical or distant metastases was proposed by Morita [38]. In an analysis of survival and prognostic factors, Jethanamest et al. applied the modified Kadish staging system to 261 cases of esthesioneuroblastoma from the SEER database [17]. Cox regression analysis results showed that the staging system was a significant predictor of disease specific survival [17]. Other staging systems, based on the TNM staging system, have been proposed by Biller and Dulguerov [39,40].

Without the benefit of a centralized pathology review we cannot stratify this SEER case series based on tumor grade; however the existing published experience and the above diagnostic considerations would support a bias toward low grade ONB reported to the SEER database [28,40] although in some studies low and high grade tumors were evenly distributed without affecting the outcome [15].

## Conclusions

This analysis of the largest series of ONB cases from the SEER database suggests, after accounting for selection bias, that best outcomes follow surgery combined with radiotherapy. The efficacy, timing, and optimum method of integrating chemotherapy with surgery and radiotherapy remain unknown.

## Competing interests

The authors declare that they have no competing interests.

## Authors' contributions

MEP and TLM reviewed the SEER database for OFN cases and performed the statistical analysis. MEP, TLM, MM, and AKS wrote the manuscript. All authors reviewed the statistical analysis results, contributed to the interpretation of the results and read and approved the final manuscript.
